# DWFS: A Wrapper Feature Selection Tool Based on a Parallel Genetic Algorithm

**DOI:** 10.1371/journal.pone.0117988

**Published:** 2015-02-26

**Authors:** Othman Soufan, Dimitrios Kleftogiannis, Panos Kalnis, Vladimir B. Bajic

**Affiliations:** 1 King Abdullah University of Science and Technology (KAUST), Computational Bioscience Research Center (CBRC), Computer, Electrical and Mathematical Sciences and Engineering Division (CEMSE), Thuwal 23955–6900, Saudi Arabia; 2 King Abdullah University of Science and Technology (KAUST), Computer, Electrical and Mathematical Sciences and Engineering Division (CEMSE), Thuwal 23955–6900, Saudi Arabia; International Centre for Genetic Engineering and Biotechnology (ICGEB), INDIA

## Abstract

Many scientific problems can be formulated as classification tasks. Data that harbor relevant information are usually described by a large number of features. Frequently, many of these features are irrelevant for the class prediction. The efficient implementation of classification models requires identification of suitable combinations of features. The smaller number of features reduces the problem’s dimensionality and may result in higher classification performance. We developed DWFS, a web-based tool that allows for efficient selection of features for a variety of problems. DWFS follows the wrapper paradigm and applies a search strategy based on Genetic Algorithms (GAs). A parallel GA implementation examines and evaluates simultaneously large number of candidate collections of features. DWFS also integrates various filtering methods that may be applied as a pre-processing step in the feature selection process. Furthermore, weights and parameters in the fitness function of GA can be adjusted according to the application requirements. Experiments using heterogeneous datasets from different biomedical applications demonstrate that DWFS is fast and leads to a significant reduction of the number of features without sacrificing performance as compared to several widely used existing methods. DWFS can be accessed online at www.cbrc.kaust.edu.sa/dwfs.

## Introduction

In the last decade, the leading high-throughput experimental techniques in biology, such as next generation sequencing, mass spectrometry, array-based methods and others, let to the massively increasing volume and dimensionality of the produced data in this field. This expansion in volume and dimensionality of data is also occurring in many other domains such as, for example, web content, social networks, graphical information systems, etc. From a Data Mining perspective, the need for faster, more reliable and more cost-effective classification models based on such data requires the extraction of a smaller and optimized set of features that can be obtained by removing largely redundant, irrelevant and unnecessary features for the class prediction [1]. A reduced number of features may also in some cases improve the classification performance [1]. In addition, the reduced number of features may lead to a better description of the underlying process from which data is generated and, thus, may contribute to better interpretation of the results [2]. Consequently, the feature selection (FS) problem is a fundamental problem for the development of efficient data-driven Machine Learning models. Although FS has a great use in many fields, we will restrict our consideration to biology and biomedical domains [3,4].

There are three basic FS methodologies: a/ the filters, b/ the wrappers, c/ and those based on the embedded FS models.

a/ Filtering approaches are mostly fast statistical methods that rank features according to specific criteria. In principle, filters do not get feedback from classification models. When the set of top ranked features is used for class prediction, classification performance is frequently inferior to the case when all features are used.

b/ In the wrapper models the process of FS is tied to the performance of a specific classification model and FS is made using some optimization methods and various search strategies [5]. A well-studied variant of the wrapper model is the randomized one, which relies on search strategies such as, for example, genetic algorithms (GA), hill climbing and simulated annealing. In these methods, through iterations, numerous heuristically selected feature subsets are evaluated based on the resultant error of the classifier. Based on the characteristics of the feature search space from where features are selected, evolutionary search methods like GA may be able to avoid to stuck in locally optimal solutions although there is no guarantees for this [5,6]. While this is an advantage, on the downside the wrapper-based FS combined with randomized search strategies is a very computationally demanding task. Also, the wrapper-based FS may lead to the selection of features biased to the classifier used in the wrapper [7,8].

c/ Embedded FS methods incorporate FS into the model development process. In the embedded FS setting, searching for the optimized feature subsets is a combined optimization process comprising selection of optimized set of features and tuning parameters of the model. A well-known embedded FS method is based on Decision Trees that has an automated FS strategy to select features according to the class discrimination capability [9].

Some of the FS methods used for biological and biomedical problems are not general enough and may be developed for specific data types (i.e., those obtained from microarrays).

With all the above-mentioned issues in mind we developed DWFS, an on-line web-based FS tool that follows the GA-based wrapper paradigm. To the best of our knowledge, there is no other FS web tool that provides: 1/ a hybrid combination of wrapper-based FS with effective filtering FS methods, 2/ parallel implementation that reduces the time required for FS, 3/ options to optimize the wrapper setting and tune weights and parameters of the FS process according to the application requirements, and 4/ applicability to a broad spectrum of applications and processing of larger multi-dimensional datasets (not only from biology and biomedicine) that existing web-based FS tools may fail to handle. DWFS is aimed for biologists that have little or no computer skills.

Our experimentation with several benchmark datasets from different biological and biomedical problems demonstrates that DWFS is capable of reducing significantly the number of original features without sacrificing classification performance. Moreover, through the use of different performance indicators we show that DWFS performs well relative to the existing FS methods.

## Materials and Methods

### Prevalent FS techniques used for biomedical problems

Previous review articles addressed the importance of selecting relevant features in biological and biomedical problems and illustrated the state-of-the–art methods [4,10,11]. From the category of wrappers, FST3 [12] and WEKA [13] offer a variety of wrapper and filtering models based on different search strategies. WEKA is widely used for its easy access and functionality. However, it is not scalable to multicore computers or high-end clusters and consequently as the data volume increases it cannot handle effectively computationally intensive FS in the wrapper setting.

From the category of filters, mRMR [14] belongs to multivariate filtering methods and a web tool is available. However, the web-tool has some limitations since only input files with size less than 2 MB are supported. FSelector [15] presents an extensive collection of existing filtering algorithms implemented in a Ruby package whereas, the CBFS algorithm [16] introduces the clearness-based scoring scheme for describing features that maximize separability between the classes.

There are also many dataset-specific FS methods, e.g. ArrayMining [17] that targets selection of genes from microarray datasets, Multi-Relief that [18] ranks crucial residues for protein functionality or MetaboloAnalyst [19] that selects important metabolites from metabolic network data. Table 1 summarizes the advantages and disadvantages of the above-mentioned FS techniques.

**Table 1:** FS programs frequently used in biomedical applications.

Tool Name	Parallel implementation	Web-interface	FS type	Reference
FST3	Yes (threading model over single machine)	no	hybrid-filter-wrapper	[12]
mRMR	no	yes	filter	[14]
Arraymining	no	yes	filter-wrapper-embedded	[17]
WEKA	no	no	filter-wrapper	[13]
Multi-Relief	no	yes	filter	[18]
MetaboloAnalyst	no	yes	filter	[19]
FSelector	no	no	filter-wrapper	[15]
CBFS	no	no	filter	[16]
FeaLect	no	no	wrapper	[35]
Proposed method DWFS	yes	yes	hybrid-filter-wrapper	

### DWFS web-tool

**Search strategy description**. DWFS takes as input text files with tab-delimited format where rows represent data samples and columns represent features. The last column represents class labels. The multiclass classification tasks (classes> = 2) are supported. The wrapper FS search strategy is based on GA that is a well-known heuristic optimization technique inspired by the principles of natural selection [6]. Initially, a starting population Θ_0_ = {θ_01_, θ_02_,…, θ_0*r*_} (the collection of initial solutions) of individuals θ_0i_,i = 1,2,…,*r*, where r is the number of individuals, is formed. The individuals represent chromosomes. Note that in our case the chromosomes are always described by vectors of length *n* whose components correspond to genes (features); *n* is the number of all features. The values of the components are binary 1s and 0s, where ‘1’ encodes the existence and ‘0’ the absence of a gene. In the initial population chromosomes are initialized by a random selection of 1s and 0s. In GA, the population changes in each evolutionary cycle forming different generations. An evolutionary cycle *k*, is characterized by the population Θ*_k_* = {θ_*k*1_, θ_*k*2_,…, θ_*kr*_}; θ*_ki_* represents the *i^th^* chromosome in population Θ*_k_*. In every evolutionary cycle, a fitness value is computed based on a predefined fitness (objective) function. DWFS uses the fitness function described by Equation (1), where *Performance*(θ*_ki_*) is the classification performance achieved by using chromosome θ*_ki_*, *size*(θ*_ki_*) is the sum of binary 1s in chromosome θ*_ki_*, and α is a weighting parameter (default 0.15).

$$\mathrm{FitnessFunction}=\underset{\Theta }{\text{max}}\left(\mathrm{Performance}\left(\theta_{ki}\right)-\alpha \times \frac{\mathrm{sum}\left(\theta_{ki}\right)}{n}\right)$$ (1)

Note that in our case the actual *Performance* function is determined by the selected criterion from the web-tool. Then, GA applies two operations, crossover and mutation, and the best solutions are selected to survive to the next generation. The optimization process is run for a specified number of evolutionary cycles, during which time GA attempts to increase the value of the fitness function. The chromosome that produces the largest value of the fitness function during this specified number of evolutionary cycles is considered an optimized solution and it may or may not be the global (optimal) solution. It is difficult to know if the obtained optimized solution is the global one. The procedure terminates whenever termination criteria are satisfied [6].

**Fig 1:**
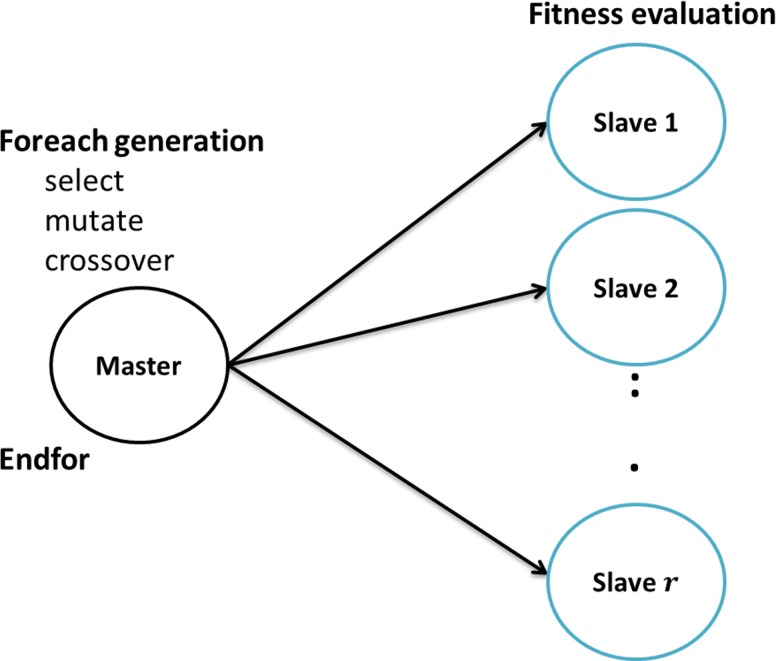
GA algorithm under master/slave framework.

**Implementation**. DWFS search strategy is based on the PGAPack software [20], which is a collection of libraries that execute all GA steps. PGAPack deploys a parallel master/slave single population GA using the message-passing interface (MPI). Master node stores the initial population and applies the GA operations. Slave nodes are responsible for evaluating the fitness function for every chromosome. The master/slave paradigm is very efficient in terms of execution time because the most costly part is the fitness function evaluation, whereas the communication overhead is minimal [21]. Fig. 1 demonstrates the GA algorithm under the master/slave framework of PGAPack. In principle, increasing the population size does not necessarily improve the classification performance as there is no guarantee that the algorithm will be able to find any better solution than it does with the original population size. We use a population size of 100 individuals, which is a good compromise considering the trade-off between the performance and over-fitting [22]. Note, that DWFS master/slave model was deployed on a cluster with nodes having 64-cores. In our experimentation in one experiment 63 cores are used for replacing the old solutions for every new generation and the 64^th^ core (i.e. the master) was responsible only for executing the GA operators. In the first population we initialize genes to 0 and 1 randomly with equal probability and we ensure that a chromosome containing all genes set to 1 is present (i.e., this represents the solution that contains all features). In our implementation we adopt constant rates for crossover and mutation. The crossover operator produces new offspring by combining genes of two chromosomes (they are considered parents in the GA terminology). We use two-point crossover technique that swaps all genes between two crossover points for both chromosomes. The following example illustrates the two-point crossover operation over a pair of chromosomes with 9 genes.

Chromosome1 (Parent1): [**0** **0** 1 1 0 1 1 **0** **1**]

Chromosome2 (Parent2): [**1** **1** 1 0 0 0 1 **1** **1**]

Two-point crossover operator ↓

Chromosome1 (Child1): [0 0 **1 0 0 0 1** 0 1]

Chromosome2 (Child2): [1 1 **1 1 0 1 1** 1 1]

The underlined features in the chosen parent solutions indicate the points within which crossover will take place (i.e. genes between these points will be crossed-over to generate child solutions). A pre-defined crossover rate will determine the number of genes where crossover will take place.

Mutation modifies specific genes of a given chromosome. We use the simple bit-string mutation that flips specific genes at random positions. Both GA operations simulate parts of the natural evolutionary process and are used to control transfer of information between different generations of population. The evolution of the population follows a binary tournament selection, where the best two chromosomes in the population are selected to serve as parents of new offspring. The GA operators including selection, mutation and crossover are then carried over in an iterative process through a predefined number of generations.

**Fig 2:**
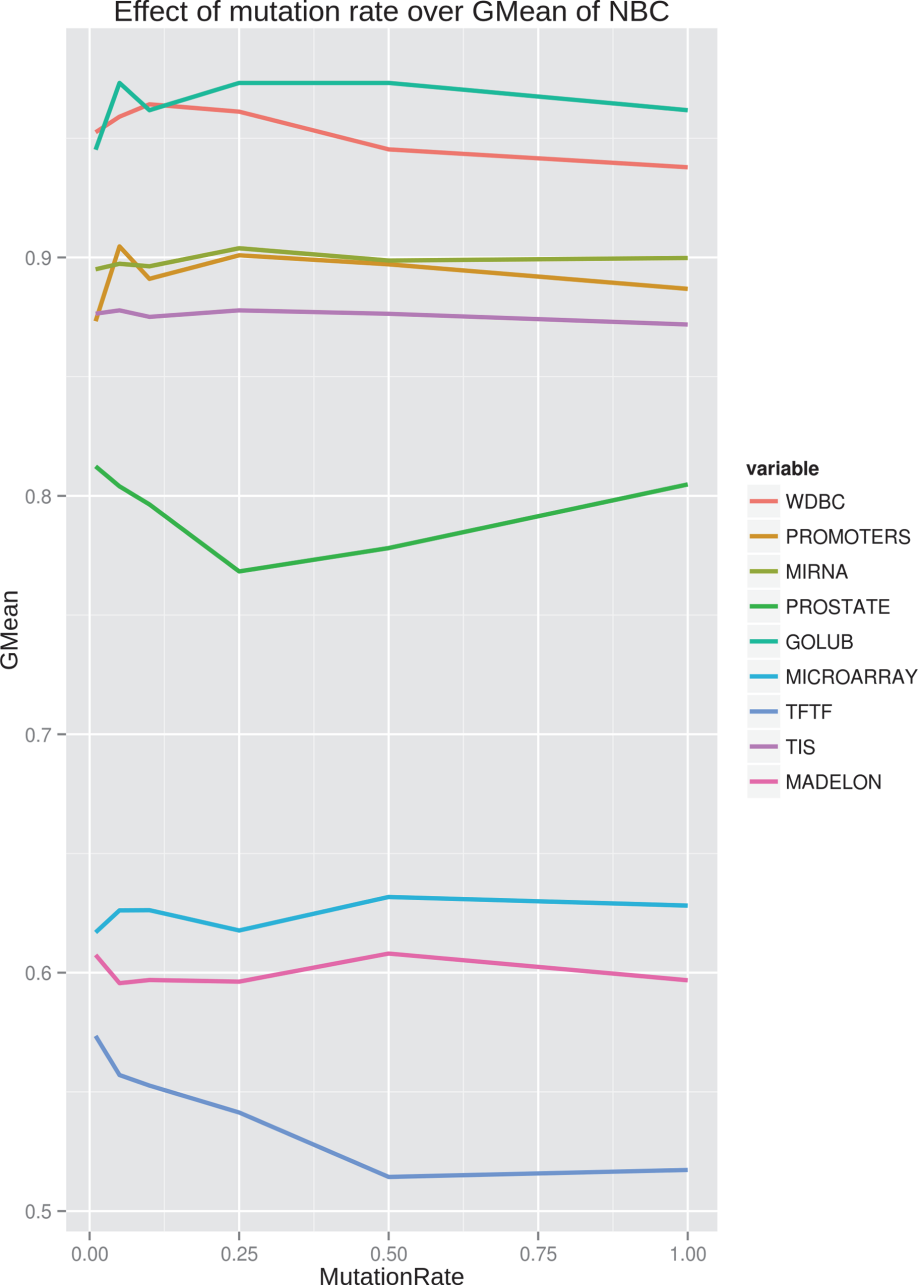
Effects of changing mutation rate on GMean of NBC. For every mutation rate on each dataset, a full run of GA is executed for 5 times to compute scores over the 5-folds of the dataset. The specific used mutation rates are 0.01, 0.05, 0.1, 0.25, 0.5, 1.

**Fig 3:**
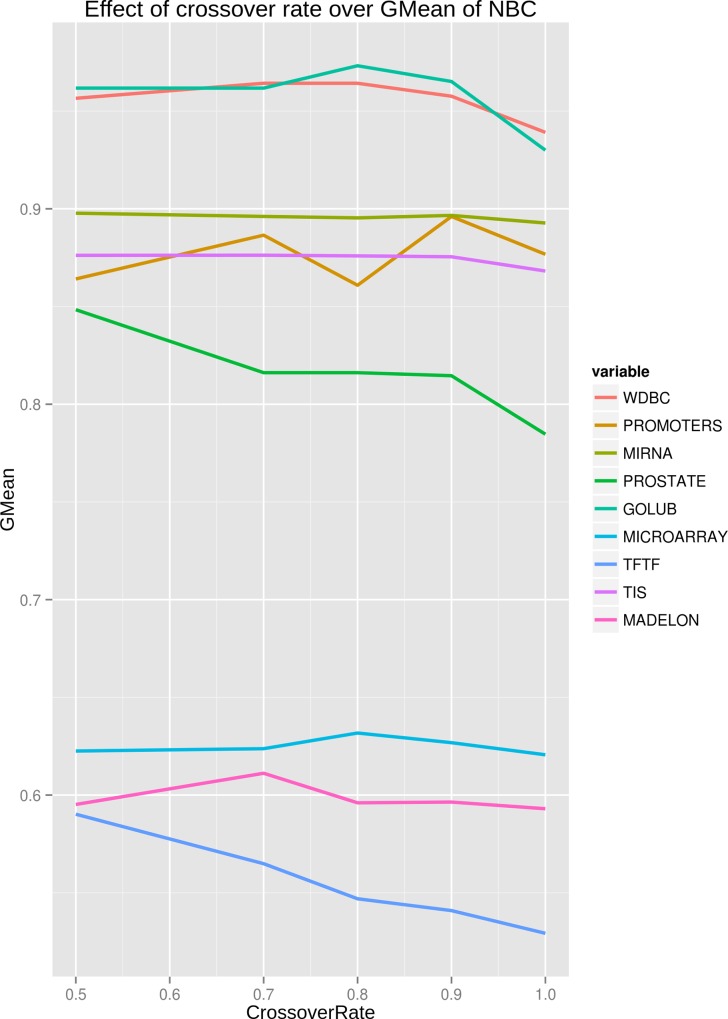
Effects of changing crossover rate on GMean of NBC. For every mutation rate on each dataset, a full run of GA is executed for 5 time to compute scores over the 5-folds of the dataset. The specific applied crossover rates are 0.5, 0.7, 0.8, 0.9, 1.

**Tuning search strategy parameters**. Depending on the application requirements users can specify mutation and crossover rates, as well as the number of generations for the optimization procedure. After experimentation with different mutation and crossover rates we observed that values close to 0.8 for crossover and 0.01 for mutation are effective in various classification tasks (see also [5]). These values we selected as default for DWFS. The effects of extensive experimentation with various mutation and crossover rates for all datasets are captured in Fig. 2 and Fig. 3. Both Fig. 2 and Fig. 3, indicate a considerable sensitivity of GA towards mutation and crossover rates. Overall, increasing either one of these rates leads to not exploiting surrounding regions of good solutions and mostly exploring the search space in a random manner. This is apparent in Fig. 2 and Fig. 3 for most of the datasets. More results about the effects of mutation and crossover rates over stability and reduction in size of selected features are provided in S1 Table and S2 Table. The default number of evolutionary cycles in DWFS is 100. Also, in order to reduce the processing time we included additional stopping criteria in which we consider that the population has reached the steady-state (i.e. it has converged) if there is no difference in the fitness function value for 50 consecutive generations.

**Modifying the fitness function**. DWFS supports different validation methods and users can choose between automatic split of data to training and testing sets, or the custom option in which they can upload explicitly the training and testing datasets. DWFS offers different performance metrics for the fitness function from Equation (1) including accuracy, geometric mean of sensitivity and specificity (GMean), F1-score and Mathews Correlation Coefficient (MCC). Possibility to select different performance metrics for the optimization process increases the utility of DWFS. For instance, GMean is preferable in cases where the data classes are highly imbalanced [23]. Three different classification models can be used: a/ Naïve Bayes Classifier (NBC-in-house implementation), b/ K-Nearest-Neighbors (KNN) available from the AlgLib library (http://www.alglib.net/) [24], and c/ and the combination of a/ and b/. In principle, selecting more than one classifier tends to minimize potential bias towards a particular type of classifier. The idea behind this is relatively simple: features selected using a single classifier in the wrapper FS usually result in higher performance for that particular classification model and weaker performance for other classifiers. Including a combination of classifiers in the wrapper FS setting, may lead to the selection of a feature subset that is less biased, but this is not guaranteed.

**Hybrid combination of filtering and wrapping**. When the number of features is very large, the GA becomes very computationally demanding and its run time can be prohibitive. To reduce the time required for training, DWFS supports a two-phase hybrid combination of filtering and wrapping. In the first phase, filtering is applied as a pre-processing step and the top-ranked features are selected based on a pre-defined but tunable threshold. In the second phase, the GA-based wrapper FS is applied to the selected features, which in practice means that the FS process uses a much smaller search space instead of the original large search space. This heuristic optimization technique may or may not result in combinations of features that have high discriminative power. However, this cannot be known in advance. In practice, however, the experiments show that this heuristics performs well and that it is fast and memory efficient for datasets described by large number of features and having large number of samples. Of course, the user may choose not to use filtering at all. Current implementation of DWFS incorporates the following well-known and widely-used filtering techniques: minimum redundancy maximum relevance (mRMR) [14], joint mutual information (JMI) [25], conditional mutual information maximization (CMIM) [26] and interaction capping (ICAP) [27]. mRMR selects features based on the minimum redundancy-maximum relevancy criterion. Both JMI and CMIM are based on different notions of mutual information (joint vs conditional), whereas ICAP selects features based on the concept of “interaction” of the class label with different sets of features. Recently, a study has proposed a unifying framework for information theoretic feature selection that derive the previous criteria from conditional likelihood of the training labels [28]. In particular, the scoring criteria including mRMR, JMI, CMIM and ICAP can be derived from the following function *F* described by Equation (2).

$$F=E_{xy}\left\{\text{log}\frac{p\left(y|x_{\theta }\right)}{q\left(y|x_{\theta },\tau \right)}\right\}+I\left(X_{\tilde{\theta }};Y|X_{\theta }\right)+H\left(Y|X\right)$$ (2)

The first term is a likelihood ratio between the true distribution *p*(*y*|*x*_θ_ of a class given some particular selected features *x*_θ_ and the predicted class distribution *q*(*y*|*x*_θ_, τ) given the selected features and a model parameter τ, averaged over the input space. Given the selected features *X*_θ_, $I\left(X_{\tilde{\theta }};Y|X_{\theta }\right)$ defines conditional mutual information between the class label *Y* and the unselected features $X_{\tilde{\theta }}$. The final term *H*(*Y*|*X*) is the conditional entropy of labels *Y* given all original set of features *X*. In [28] a rigorous derivation of many information theoretic measures for feature selection from Equation (2) is provided to eventually reveal either a direct or conditionally dependent relationship between the target label and the selected features of dataset.

## Results and Discussion

### Experimental setup

**Datasets**. In our experiments we use nine datasets related to different biomedical problems. Table 2 summarizes the number of samples, number of features, ratio between positive and negative samples and corresponding data sources. We chose on purpose datasets from a broad spectrum of different problems to demonstrate that DWFS is a general tool for FS and that it performs well over a variety of different types of data.

**Table 2:** Datasets used in our experimental setup.

Dataset name	Number of Samples	Number of features	Number of positive/negative	Reference
TIS	25604	81	12802/12802	[36]
TFTF	674	1062	339/335	[32]
Medelon	2600	500	1300/1300	[37]
Wdbc	569	30	357/212	[37]
Promoters	106	57	53/53	[37]
Pre-miRNAs	9185	48	691/8494	[38]
Lung cancer (microarrays)	187	19993	90/97	[39]
Leukemia (microarrays)	72	7129	47/25	[40]
Prostate cancer (microarrays)	102	2135	52/50	[41]

**The methods used in comparison**. To estimate the effectiveness of DWFS and quantify the contribution of the filtering (pre-processing) step, we select features using wrapper FS only (denoted as DWFS), wrapper FS combined with mRMR (denoted as mRMR+DWFS) and wrapper FS combined with JMI (denoted as JMI+DWFS). To provide better cross-benchmarking results, we compare the classification performance of DWFS and its variants with the most effective wrappers and the filtering approaches presented in Table 1. Specifically, we include in the comparison WEKA (version 3.6.6) wrapper that uses an integrated method of forward and backward search (Bi-directional BestFirst), FST3 that implements a powerful wrapper-based method called sequential forward floating search (SFFS), as well as mRMR and JMI filters. In our comparison analysis, we view WEKA and FST3 wrappers as representative implementations for other tools that use similar sophisticated approaches for FS. Moreover, mRMR and JMI filters are also considered representatives of a wider class of advanced filtering approaches. In particular, JMI stands as a representative for single-objective filtering methods that examine joint relevance between features with the target variable, while mRMR represent methods that consider multi-objective formulation for filtering features with high relevance to the target and minimum redundancy between each other. In other words, comparison with WEKA and FST3 wrappers and mRMR and JMI filters, is expected to reveal how our approach may compare to other filters or wrappers. We used two baselines, one where the classification performance is obtained utilizing all features (the initial/original feature vector), and the other that uses top 10% of features with the highest class correlation (we call it Correlation-baseline). We did not include in the comparison the dataset-specific FS tools such as Arraymining, Multi-Relief and MetaboloAnalyst, or tools that at the time of this study were not fully functional (e.g. that crushed during experiments).

**Performance assessment**. Classification performance is assessed using nested 5-fold cross-validation. This technique splits data to five folds and for every fold FS is applied. To evaluate the performance of the model inside a single fold we split again this particular fold to five folds, assign the performances and average the classification performance of the folds. Such cross-validation setup has been proposed in [29] for avoiding potential over-fitting effects. Given the large size of our experimental datasets, 5-fold setup seems to be a proper choice for computing a representative (i.e. not biased) estimate while being computationally faster compared to a setup with a larger number of folds [30,31].

Classification performance is measured using five classification performance metrics, namely: Sensitivity that measures the true positive rate; Specificity that measures true negative rate; GMean that measures the combined effect of Sensitivity and Specificity; Positive Predictive Value (PPV) that measures proportion of correctly predicted positives out of all predicted positives; and F1-measure that estimates the combined effect of PPV and Sensitivity. These performance metrics are frequently used performance indicators for classification systems [23,32]. Let TP be the number of true positives, FP the number of false positives, TN the number of true negatives and FN the number of false negatives, Equations 3 to 7 show formulae of these performance metrics:

Sensitivity=TP/(TP+FN) (3)

Specificity=TN/(TN+FP) (4)

PPV=TP/(TP+FP) (5)

$$\mathrm{GMean}=\sqrt{\mathrm{sensitivity}\times \mathrm{specificity}}$$ (6)

$$F_{1}=2\times \frac{\mathrm{precision}\times \mathrm{sensitivity}}{\mathrm{precision}+\mathrm{sensitivity}}$$ (7)

In addition to classification performance indicators, we also include a measure of robustness called stability [33]. The stability *S*(*θ_i_*, *θ_j_*) between two selected feature subsets *θ_i_*, *θ_j_* over two different folds of the data is defined by Equation (8). The sizes of these two feature subsets are represented by *k_i_*, *k_j_*, respectively. The size of common features (i.e. the number of elements in the intersection between the two sets) is *c*:

$$S\left(\theta_{i},\theta_{j}\right)=\frac{c-\frac{k_{i}k_{j}}{n}}{\text{min}\left(k_{i},k_{j}\right)-\text{max}\left(0,k_{i}+k_{j}-n\right)}$$ (8)

We compute the pair-wise scores in Equation (8) for different folds (i.e. 5 folds in our setup) and report their average. The stability measure in Equation (8) has a range of (−1,1] were a value of 0 indicates an output from a random FS. A positive range states that the FS method is more stable than a random one and a negative value indicate the opposite [33].

### Comparison analysis

**Classification performance and ranking**. We do not know in advance the specifics of the classifier implementation that the different tools have. For this reason, we select features with each of the wrapper-based tools included in the comparison, using their in-house implementations. However, we evaluate all programs using the same MATLAB R2012b implementations as these were not part of any of the wrapper-based FS tools used in the comparison analysis. This ensures fairness in the comparison. Finally, for JMI, mRMR and Correlation-baseline, we select the 10% of the top-ranked features.

Tables 3–20 present the actual average and standard deviation of classification performance for all studied methods using NBC and KNN classifiers. We observe that, using different performance indicators the studied programs show different advantages and disadvantages and perform differently on different datasets. In terms of GMean, DWFS is comparable in many cases to the other methods. For the KNN classifier, in 6 out of 9 datasets, DWFS hybrid with a filtering method enhanced the filtering performance. This, nevertheless, is not the case for the NBC classifier where such enhancement happens in 4 out of 9 datasets. In 3 out of 18 cases (9 datasets with 2 classifiers), WEKA achieved the best results with the NBC and KNN classifiers. The same was also the case for FST3. Interestingly, the Correlation-baseline achieved the best result for the Microarray dataset with the KNN classifier (Table 16). These experimental results suggest that DWFS or one of its variants are better suited for datasets with large number of samples (>200).

**Table 3:** Actual classification performance for WDBC dataset using KNN classifier.

	Sensitivity	Specificity	GMean	PPV	F1-measure
DWFS	96.86% (±2.68)	89.38% (±4.49)	93% (±1.71)	93.7% (±3.57)	95.2% (±1.89)
MRMR+DWFS	91.38% (±5.71)	85.61% (±5.07)	88.32% (±0.98)	91.35% (±3.92)	91.19% (±2.27)
JMI+DWFS	91.38% (±5.71)	85.61% (±5.07)	88.32% (±0.98)	91.35% (±3.92)	91.19% (±2.27)
MRMR	91.66% (±5.21)	85.61% (±5.07)	88.47% (±0.96)	91.37% (±3.93)	91.36% (±2.06)
JMI	95.31% (±3.26)	88.44% (±3.25)	91.8% (±2.61)	93.41% (±1.35)	94.33% (±1.88)
WEKA	92.2% (±3.95)	86.53% (±2.73)	89.29% (±2.12)	91.8% (±3.03)	91.93% (±2.34)
FST3	95.5% (±3.19)	87.92% (±5.9)	91.57% (±3.07)	92.9% (±4.29)	94.11% (±2.49)
ALL Features	96.16% (±3.59)	88.57% (±3.64)	92.27% (±3.06)	93.43% (±1.97)	94.75% (±2.3)
Correlation-baseline	91.3% (±5.48)	84.05% (±5.3)	87.52% (±3.63)	90.19% (±5)	90.61% (±3.86)

**Table 4:** Actual classification performance for Promoter dataset using KNN classifier.

	Sensitivity	Specificity	GMean	PPV	F1-measure
DWFS	98% (±4.47)	71.4% (±7.4)	83.54% (±4.52)	75.05% (±13.13)	84.46% (±8.65)
MRMR+DWFS	90.96% (±10.28)	74.5% (±9.86)	82.11% (±7.38)	78.42% (±9.82)	83.39% (±4.64)
JMI+DWFS	88.74% (±11.91)	72.83% (±6.29)	80.16% (±5.8)	76.19% (±7.94)	81.17% (±3.95)
MRMR	91.24% (±9.34)	79.51% (±7.48)	85.04% (±6.74)	81.94% (±4.17)	86.13% (±4.93)
JMI	91.24% (±9.34)	82.84% (±11.97)	86.77% (±8.85)	81.62% (±13.06)	85.74% (±9.17)
WEKA	87.41% (±12.69)	76.55% (±8.85)	81.48% (±7.61)	76.71% (±14.39)	81.05% (±12.04)
FST3	85.42% (±13.88)	74.5% (±5.27)	79.33% (±4.45)	75.44% (±11.12)	78.9% (±6.8)
ALL Features	85.89% (±11.57)	82.84% (±2.11)	84.21% (±6.32)	82.62% (±5.33)	83.56% (±3.48)
Correlation-baseline	82.2% (±17.77)	75.37% (±13.6)	78.19% (±12.17)	75.3% (±17.67)	76.96% (±12.89)

**Table 5:** Actual classification performance for miRNA dataset using KNN classifier.

	Sensitivity	Specificity	GMean	PPV	F1-measure
DWFS	71.79% (±6.3)	98.65% (±0.39)	84.09% (±3.68)	81.07% (±3.64)	76.02% (±4.1)
MRMR+DWFS	69.77% (±6.93)	98.03% (±0.18)	82.62% (±4.11)	74.07% (±2.71)	71.74% (±4.38)
JMI+DWFS	68.49% (±5.81)	97.99% (±0.1)	81.86% (±3.41)	73.25% (±2.26)	70.69% (±3.37)
MRMR	68.27% (±7.79)	98.88% (±0.32)	82.06% (±4.64)	83.34% (±2.88)	74.91% (±5.32)
JMI	57.49% (±6.82)	99.2% (±0.24)	75.41% (±4.47)	85.31% (±4.29)	68.53% (±5.51)
WEKA	67.01% (±16.86)	99.22% (±0.34)	81.03% (±10.46)	86.56% (±8.08)	75.12% (±13.62)
FST3	78.24% (±6.73)	99.41% (±0.16)	88.13% (±3.79)	91.58% (±1.67)	84.25% (±3.96)
ALL Features	59.78% (±7.61)	99.52% (±0.26)	77.01% (±4.77)	91.32% (±3.73)	71.98% (±5.18)
Correlation-baseline	70.12% (±7.14)	98.56% (±0.3)	83.05% (±4.23)	79.7% (±4.37)	74.46% (±4.96)

**Table 6:** Actual classification performance for Prostate cancer dataset using KNN classifier.

	Sensitivity	Specificity	GMean	PPV	F1-measure
DWFS	87.56% (±4.9)	85.33% (±13.44)	86.17% (±7.41)	84.57% (±13.5)	85.59% (±8.06)
MRMR+DWFS	94.42% (±5.24)	92.05% (±7.95)	93.17% (±5.68)	92.64% (±8.23)	93.4% (±5.83)
JMI+DWFS	98.18% (±4.07)	86.47% (±7.9)	92.03% (±4.47)	87.44% (±7.21)	92.3% (±4.21)
MRMR	88.93% (±10.55)	88.41% (±14.91)	87.99% (±5.99)	90.31% (±12.61)	88.54% (±5.78)
JMI	85.8% (±5.23)	84.65% (±11.89)	85.14% (±8.44)	84.39% (±11.83)	84.92% (±8.58)
WEKA	75.83% (±19.09)	83.51% (±17.2)	79.29% (±16.66)	79.85% (±20.81)	77.48% (±19.07)
FST3	84.48% (±9.33)	84.12% (±10.11)	84% (±6.19)	83.63% (±9.01)	83.88% (±8.16)
ALL Features	79.58% (±10.6)	83.11% (±11.12)	81.2% (±9.68)	81.02% (±13.52)	79.97% (±10.86)
Correlation-baseline	92.25% (±7.47)	87.15% (±9.92)	89.44% (±5.41)	86.52% (±11.1)	88.84% (±6.53)

**Table 7:** Actual classification performance for Leukemia dataset using KNN classifier.

	Sensitivity	Specificity	GMean	PPV	F1-measure
DWFS	97.78% (±4.97)	85% (±22.36)	90.32% (±12.26)	91.92% (±11.57)	94.3% (±6.03)
MRMR+DWFS	94.29% (±5.31)	45.5% (±33.65)	61.74% (±22.79)	74.16% (±19.12)	81.57% (±11.83)
JMI+DWFS	90.81% (±8.79)	90.17% (±9.36)	90.18% (±3.47)	92.92% (±6.65)	91.37% (±2.26)
MRMR	92.78% (±7.24)	23% (±22.8)	35.98% (±33.43)	69.88% (±18.13)	78.87% (±15.03)
JMI	89.29% (±11.15)	68.67% (±22.19)	77.39% (±13.67)	81.72% (±14.64)	84.42% (±9.83)
WEKA	96.52% (±4.78)	86.17% (±16.41)	90.68% (±7.6)	91.51% (±8.28)	93.59% (±2.39)
FST3	95.96% (±5.58)	75.17% (±14.79)	84.47% (±8.7)	89.56% (±2.49)	92.57% (±2.87)
ALL Features	97.78% (±4.97)	82.67% (±10.31)	89.76% (±6.26)	89.11% (±9.01)	93% (±5.68)
Correlation-baseline	98.33% (±3.73)	90% (±13.69)	93.79% (±6.79)	93.33% (±10.87)	95.4% (±5.84)

**Table 8:** Actual classification performance for Lung cancer dataset using KNN classifier.

	Sensitivity	Specificity	GMean	PPV	F1-measure
DWFS	63.52% (±10.21)	66.39% (±13.29)	64.82% (±10.73)	63.49% (±17.41)	63.14% (±13.27)
MRMR+DWFS	61.82% (±10.6)	66.72% (±6.37)	64.1% (±7.61)	62.88% (±9.13)	61.94% (±7.66)
JMI+DWFS	56.14% (±16.39)	78.33% (±8.16)	65.65% (±10.19)	70.06% (±13.39)	61.27% (±12.33)
MRMR	57.38% (±10.61)	71.94% (±13.29)	63.98% (±9.83)	66.19% (±12.92)	60.84% (±9.39)
JMI	54.96% (±16.17)	72.17% (±11.17)	62.43% (±11.93)	64.29% (±17.51)	58.41% (±14.32)
WEKA	61.66% (±7.95)	63.06% (±11.61)	62.04% (±7.44)	60.8% (±14.68)	60.26% (±9.33)
FST3	57.63% (±9.72)	67.06% (±5.66)	61.82% (±4.75)	61.37% (±9.92)	58.88% (±8.14)
ALL Features	59.43% (±12.14)	63.67% (±17.65)	60.89% (±10.61)	61.62% (±18.05)	59.65% (±12.32)
Correlation-baseline	60.29% (±8.75)	65.44% (±10.5)	62.5% (±6.47)	61.44% (±12.68)	60.19% (±7.55)

**Table 9:** Actual classification performance for TFTF dataset using KNN classifier.

	Sensitivity	Specificity	GMean	PPV	F1-measure
DWFS	78.23% (±6.39)	57.12% (±6.51)	66.82% (±6.34)	64.79% (±7.13)	70.86% (±6.9)
MRMR+DWFS	80.68% (±5.59)	57.53% (±7.49)	67.97% (±5.07)	65.87% (±5.05)	72.46% (±4.79)
JMI+DWFS	76.18% (±2.41)	60.34% (±9.12)	67.66% (±5.66)	66.39% (±4.12)	70.9% (±2.81)
MRMR	74.96% (±4.83)	57.6% (±9.61)	65.48% (±5.72)	64.35% (±7.18)	69.02% (±4.83)
JMI	76.29% (±5.13)	56.9% (±6.39)	65.83% (±5.2)	64.11% (±6.68)	69.6% (±5.65)
WEKA	77.46% (±6.4)	56.57% (±5.77)	66.1% (±4.91)	64.31% (±5.14)	70.13% (±4.57)
FST3	78.75% (±2.22)	58.38% (±5.02)	67.76% (±3.38)	65.66% (±5.29)	71.53% (±3.65)
ALL Features	75.98% (±5.78)	56.35% (±6.79)	65.36% (±5.53)	63.71% (±7.1)	69.22% (±6.09)
Correlation-baseline	76.23% (±4.36)	55.14% (±7.5)	64.74% (±5.53)	63.25% (±7)	69.05% (±5.7)

**Table 10:** Actual classification performance for TIS dataset using KNN classifier.

	Sensitivity	Specificity	GMean	PPV	F1-measure
DWFS	84.02% (±0.87)	85.62% (±1.38)	84.81% (±0.73)	85.4% (±1.24)	84.7% (±0.74)
MRMR+DWFS	95.38% (±10.34)	15.82% (±35.38)	15.6% (±34.88)	55.71% (±12.89)	68.86% (±5.02)
JMI+DWFS	88.85% (±10.18)	49.81% (±45.47)	49.33% (±45.03)	69.66% (±17.85)	75.94% (±8.38)
MRMR	82.18% (±0.92)	82.35% (±1.02)	82.26% (±0.9)	82.32% (±1.02)	82.25% (±0.94)
JMI	83.85% (±0.81)	85.32% (±0.51)	84.58% (±0.49)	85.1% (±0.65)	84.47% (±0.64)
WEKA	83.74% (±0.45)	84.79% (±1.29)	84.26% (±0.59)	84.65% (±0.96)	84.19% (±0.43)
FST3	83.8% (±0.5)	85.37% (±1.62)	84.58% (±1.01)	85.15% (±1.5)	84.46% (±0.95)
ALL Features	85.66% (±0.34)	85.29% (±0.71)	85.47% (±0.26)	85.34% (±0.69)	85.5% (±0.31)
Correlation-baseline	81.82% (±0.47)	81.82% (±1.23)	81.82% (±0.44)	81.83% (±0.92)	81.82% (±0.34)

**Table 11:** Actual classification performance for Medelon dataset using KNN classifier.

	Sensitivity	Specificity	GMean	PPV	F1-measure
DWFS	77.99% (±2.05)	79.33% (±4.47)	78.63% (±2.58)	78.99% (±5.24)	78.44% (±3.28)
MRMR+DWFS	53.01% (±4.15)	51% (±4.03)	51.9% (±1.88)	51.97% (±3.52)	52.35% (±2.51)
JMI+DWFS	88.53% (±2.18)	89.69% (±1.71)	89.09% (±0.74)	89.56% (±1.73)	89.01% (±1.07)
MRMR	49.16% (±1.3)	54.82% (±2.38)	51.9% (±1.21)	52.12% (±3.74)	50.54% (±1.98)
JMI	76.91% (±2.89)	76.78% (±3.68)	76.79% (±0.71)	76.81% (±3.9)	76.75% (±1.27)
WEKA	87.92% (±2.03)	88.36% (±1.67)	88.12% (±0.45)	88.24% (±2.3)	88.04% (±0.55)
FST3	89.28% (±2.17)	89.79% (±0.69)	89.52% (±0.93)	89.67% (±1.57)	89.45% (±1.05)
ALL Features	68.8% (±4.61)	72.99% (±4.27)	70.79% (±2.43)	71.84% (±4.04)	70.19% (±3.35)
Correlation-baseline	81.18% (±2.27)	82.7% (±1.71)	81.93% (±1.18)	82.41% (±2.09)	81.75% (±0.51)

**Table 12:** Actual classification performance for WDBC dataset using NBC classifier.

	Sensitivity	Specificity	GMean	PPV	F1-measure
DWFS	97.15% (±2.2%)	93.67% (±2.55%)	95.39% (±2.05%)	95.99% (±2.39%)	96.55% (±1.93%)
MRMR+DWFS	97.78% (±2.57%)	80.87% (±3.73%)	88.91% (±2.65%)	89.38% (±3.68%)	93.35% (±2.48%)
JMI+DWFS	97.78% (±2.57%)	80.87% (±3.73%)	88.91% (±2.65%)	89.38% (±3.68%)	93.35% (±2.48%)
MRMR	95.53% (±1.68%)	89.36% (±8.57%)	92.29% (±3.94%)	93.4% (±5.83%)	94.36% (±2.73%)
JMI	97.48% (±2.19%)	80.35% (±4.13%)	88.47% (±2.2%)	89.14% (±3.41%)	93.08% (±1.97%)
WEKA	98.11% (±1.2%)	92.4% (±4.14%)	95.18% (±1.65%)	95.28% (±3.38%)	96.63% (±1.25%)
FST3	94.97% (±3.62%)	82.21% (±6.1%)	88.26% (±2.09%)	89.92% (±3.51%)	92.29% (±1.62%)
ALL Features	95.02% (±3.69%)	90.53% (±5.63%)	92.67% (±2.09%)	94.13% (±4.1%)	94.48% (±1.82%)
Correlation-baseline	96.59% (±1.39%)	92.84% (±4.99%)	94.67% (±2.65%)	95.45% (±3.54%)	95.99% (±2.06%)

**Table 13:** Actual classification performance for Promoters dataset using NBC classifier.

	Sensitivity	Specificity	GMean	PPV	F1-measure
DWFS	86.07% (±14.78%)	91.09% (±7.17%)	88.23% (±8.54%)	89.46% (±7.19%)	87.03% (±8.04%)
MRMR+DWFS	85.38% (±15.35%)	86.9% (±10.1%)	85.83% (±10.13%)	83.29% (±14.33%)	83.52% (±11.53%)
JMI+DWFS	85.38% (±15.35%)	86.9% (±10.1%)	85.83% (±10.13%)	83.29% (±14.33%)	83.52% (±11.53%)
MRMR	86.52% (±15.08%)	91.09% (±7.17%)	88.35% (±7.57%)	89.49% (±6.49%)	87.25% (±7.96%)
JMI	88.74% (±11.91%)	91.09% (±7.17%)	89.61% (±5.77%)	89.85% (±6.28%)	88.72% (±5.4%)
WEKA	85.95% (±14.19%)	86.23% (±11.87%)	85.6% (±8.34%)	84.32% (±12.07%)	84.1% (±8.29%)
FST3	66.38% (±27.27%)	77.84% (±7.34%)	70.92% (±17.28%)	73.75% (±8.62%)	68.13% (±17.74%)
ALL Features	87.89% (±13.21%)	96.18% (±5.24%)	91.67% (±7.08%)	96.18% (±5.24%)	91.31% (±7.53%)
Correlation-baseline	83.27% (±14.75%)	87.75% (±5.72%)	85.19% (±8.54%)	87.64% (±3.32%)	84.77% (±7.74%)

**Table 14:** Actual classification performance for miRNA dataset using NBC classifier.

	Sensitivity	Specificity	GMean	PPV	F1-measure
DWFS	83.95% (±6.3%)	96.75% (±0.7%)	90.07% (±3.18%)	67.54% (±3.18%)	74.66% (±1.34%)
MRMR+DWFS	72.71% (±7.33%)	99% (±0.22%)	84.76% (±4.25%)	85.48% (±2.92%)	78.45% (±4.87%)
JMI+DWFS	70.81% (±6.96%)	99.13% (±0.28%)	83.7% (±4.06%)	87.06% (±2.55%)	77.94% (±4.29%)
MRMR	76.65% (±5.79%)	97.61% (±0.58%)	86.45% (±3.31%)	72.38% (±3.84%)	74.38% (±4.08%)
JMI	76.93% (±5.72%)	97.82% (±0.48%)	86.71% (±3.39%)	74.09% (±5.88%)	75.44% (±5.41%)
WEKA	70.48% (±7.66%)	99.46% (±0.18%)	83.62% (±4.5%)	91.52% (±2.3%)	79.39% (±4.37%)
FST3	82.02% (±5.12%)	96.52% (±1.49%)	88.93% (±2.36%)	66.9% (±8.27%)	73.24% (±4.19%)
ALL Features	83.02% (±5.46%)	95.17% (±0.43%)	88.85% (±2.84%)	58.17% (±3.18%)	68.31% (±2.76%)
Correlation-baseline	79.01% (±6.25%)	97.4% (±0.41%)	87.67% (±3.44%)	71.08% (±3.68%)	74.71% (±3.51%)

**Table 15:** Actual classification performance for Prostate cancer dataset using NBC classifier.

	Sensitivity	Specificity	GMean	PPV	F1-measure
DWFS	76.18% (±13.41%)	85.33% (±13.44%)	80.48% (±12.13%)	82.29% (±17.43%)	78.62% (±13.39%)
MRMR+DWFS	90.78% (±11.2%)	86.19% (±11.8%)	88.06% (±7.2%)	87.37% (±9.86%)	88.33% (±6.31%)
JMI+DWFS	96.64% (±4.62%)	86.47% (±7.9%)	91.29% (±4.46%)	87.33% (±7.11%)	91.5% (±3.6%)
MRMR	89.25% (±11.46%)	85.33% (±13.44%)	86.77% (±7.7%)	85.4% (±13.15%)	86.23% (±6.91%)
JMI	90.66% (±9.71%)	85.33% (±13.44%)	87.82% (±10.48%)	84.8% (±14.25%)	87.2% (±10.32%)
WEKA	94.55% (±12.2%)	85.33% (±9.89%)	89.31% (±3.84%)	85.79% (±10.47%)	88.93% (±4.82%)
FST3	90.66% (±9.71%)	83.79% (±13.97%)	86.96% (±10.22%)	83.24% (±15.57%)	86.17% (±10.53%)
ALL Features	72.83% (±14.63%)	81.57% (±11.39%)	76.83% (±11.29%)	77.94% (±15.89%)	74.5% (±12.39%)
Correlation-baseline	90.66% (±9.71%)	85.33% (±13.44%)	87.82% (±10.48%)	84.8% (±14.25%)	87.2% (±10.32%)

**Table 16:** Actual classification performance for Leukemia dataset using NBC classifier.

	Sensitivity	Specificity	GMean	PPV	F1-measure
DWFS	100% (±0%)	90% (±22.36%)	94.14% (±13.1%)	96.92% (±6.88%)	98.33% (±3.73%)
MRMR+DWFS	84.85% (±5.19%)	58.5% (±30.9%)	68.31% (±18.71%)	77.16% (±19.03%)	79.86% (±11.78%)
JMI+DWFS	94.44% (±7.86%)	90% (±13.69%)	91.99% (±9.08%)	92.62% (±12.38%)	93.34% (±9.54%)
MRMR	83.43% (±6.6%)	79.17% (±31.18%)	79.93% (±21.17%)	88.99% (±11.61%)	86% (±8.39%)
JMI	100% (±0%)	95% (±11.18%)	97.32% (±5.99%)	98.33% (±3.73%)	99.13% (±1.94%)
WEKA	92.22% (±7.45%)	79.17% (±18.63%)	85.01% (±12.05%)	91.44% (±5.31%)	91.58% (±3.61%)
FST3	94.29% (±5.31%)	75.17% (±14.79%)	83.86% (±9.91%)	89.44% (±2.32%)	91.7% (±2.13%)
ALL Features	100% (±0%)	95% (±11.18%)	97.32% (±5.99%)	98.33% (±3.73%)	99.13% (±1.94%)
Correlation-baseline	95.96% (±5.58%)	95% (±11.18%)	95.37% (±7.57%)	98.18% (±4.07%)	97.01% (±4.25%)

**Table 17:** Actual classification performance for Lung cancer dataset using NBC classifier.

	Sensitivity	Specificity	GMean	PPV	F1-measure
DWFS	70.88% (±7.37%)	54.72% (±8.52%)	62.25% (±7.97%)	59.12% (±12.21%)	64.14% (±10.16%)
MRMR+DWFS	60.58% (±14.92%)	67.44% (±5.47%)	63.7% (±10.32%)	62.11% (±17.08%)	61.19% (±15.72%)
JMI+DWFS	68.97% (±12.04%)	59.56% (±15.97%)	63.49% (±10.97%)	61.6% (±16.54%)	64.32% (±13.83%)
MRMR	60.58% (±14.92%)	67.44% (±5.47%)	63.7% (±10.32%)	62.11% (±17.08%)	61.19% (±15.72%)
JMI	69.21% (±12.47%)	57.44% (±13.2%)	62.55% (±9.82%)	60.09% (±15.12%)	63.64% (±13.11%)
WEKA	60.71% (±13.47%)	71.89% (±8.35%)	65.53% (±7.44%)	66.03% (±12.06%)	62.18% (±9.9%)
FST3	68.9% (±9.03%)	58.33% (±22.24%)	62.43% (±13.02%)	62.87% (±17.08%)	64.67% (±11.19%)
ALL Features	71.75% (±6.77%)	54.89% (±10.12%)	62.68% (±8.58%)	59.62% (±13.21%)	64.78% (±10.94%)
Correlation-baseline	66.69% (±11.35%)	66.5% (±11.37%)	66.32% (±8.98%)	64.44% (±15.52%)	65.18% (±12.51%)

**Table 18:** Actual classification performance for TFTF dataset using NBC classifier.

	Sensitivity	Specificity	GMean	PPV	F1-measure
DWFS	84.7% (±2.79%)	36.91% (±4.4%)	55.82% (±3.24%)	57.59% (±4.43%)	68.46% (±3.2%)
MRMR+DWFS	74.99% (±4.17%)	57.44% (±6.27%)	65.49% (±3.14%)	64.22% (±1.47%)	69.11% (±1.15%)
JMI+DWFS	76.76% (±4.84%)	48.09% (±3.91%)	60.69% (±3.17%)	59.91% (±3.64%)	67.22% (±3.27%)
MRMR	91.2% (±3.97%)	24.09% (±7.09%)	46.4% (±6.83%)	54.89% (±4.63%)	68.41% (±3.72%)
JMI	88.03% (±2.76%)	21.09% (±6.9%)	42.62% (±6.7%)	53.07% (±4.18%)	66.09% (±2.82%)
WEKA	77.47% (±7.48%)	57.7% (±6.58%)	66.6% (±2.99%)	64.95% (±4.85%)	70.49% (±4.45%)
FST3	81.18% (±3.68%)	37.24% (±10.25%)	54.47% (±6.35%)	56.88% (±5.41%)	66.68% (±3.03%)
ALL Features	90.08% (±3.98%)	26.16% (±8.03%)	48% (±7.45%)	55.3% (±4.51%)	68.39% (±3.23%)
Correlation-baseline	90.01% (±4.99%)	25.11% (±10.71%)	46.34% (±10.08%)	54.99% (±5.4%)	68.07% (±4.06%)

**Table 19:** Actual classification performance for TIS dataset using NBC classifier.

	Sensitivity	Specificity	GMean	PPV	F1-measure
DWFS	87.43% (±0.57%)	88.03% (±0.66%)	87.73% (±0.4%)	87.96% (±0.53%)	87.69% (±0.31%)
MRMR+DWFS	78.31% (±0.53%)	88.22% (±0.49%)	83.12% (±0.43%)	86.93% (±0.29%)	82.4% (±0.39%)
JMI+DWFS	78.31% (±0.53%)	88.22% (±0.49%)	83.12% (±0.43%)	86.93% (±0.29%)	82.4% (±0.39%)
MRMR	85.14% (±0.57%)	87.3% (±0.53%)	86.21% (±0.24%)	87.03% (±0.35%)	86.07% (±0.23%)
JMI	82.85% (±0.75%)	86.83% (±0.68%)	84.81% (±0.46%)	86.29% (±0.41%)	84.53% (±0.42%)
WEKA	87.46% (±0.43%)	88.26% (±0.52%)	87.86% (±0.33%)	88.17% (±0.37%)	87.81% (±0.28%)
FST3	81.71% (±2.45%)	82.8% (±1.73%)	82.25% (±1.99%)	82.6% (±1.86%)	82.15% (±2.12%)
ALL Features	83.57% (±0.8%)	85.5% (±0.85%)	84.53% (±0.43%)	85.22% (±0.62%)	84.39% (±0.45%)
Correlation-baseline	86.78% (±0.41%)	86.47% (±0.69%)	86.62% (±0.39%)	86.52% (±0.52%)	86.65% (±0.28%)

**Table 20:** Actual classification performance for Medelon dataset using NBC classifier.

	Sensitivity	Specificity	GMean	PPV	F1-measure
DWFS	59.49% (±3.11%)	61.43% (±4.7%)	60.4% (±2.65%)	60.72% (±4.01%)	59.99% (±2.19%)
MRMR+DWFS	50.76% (±8.85%)	55.93% (±11.8%)	52.49% (±1.58%)	53.95% (±4.36%)	51.71% (±4.01%)
JMI+DWFS	58.47% (±5.52%)	62.84% (±6.6%)	60.41% (±2.23%)	61.31% (±3.95%)	59.6% (±2.11%)
MRMR	52.63% (±1.12%)	54.8% (±5.27%)	53.66% (±2.76%)	53.9% (±5.59%)	53.14% (±2.9%)
JMI	58.59% (±1.57%)	61.59% (±4.89%)	60.04% (±2.69%)	60.48% (±4.57%)	59.44% (±2.27%)
WEKA	58.21% (±3.98%)	63.57% (±6.1%)	60.7% (±2.4%)	61.65% (±4.6%)	59.68% (±1.93%)
FST3	57.38% (±5.38%)	62.14% (±4.68%)	59.58% (±2.32%)	60.28% (±2.97%)	58.59% (±2.03%)
ALL Features	57.57% (±1.53%)	59.48% (±5.36%)	58.47% (±2.84%)	58.79% (±5.04%)	58.07% (±2.6%)
Correlation-baseline	57.02% (±3.23%)	62.78% (±5.23%)	59.77% (±2.91%)	60.57% (±5.11%)	58.63% (±3.2%)

To draw conclusions about their relative performance, we rank the FS methods following the method presented in [34] as follows: for every dataset and for every performance indicator, the FS method that achieves the best result is ranked first and obtains 1 point, the second best obtains 2 points and so on, up to 9 points. The overall ranking for each FS method is reflected in the sum of obtained points across all datasets and all performance metrics using KNN and NBC, which we call the final ranking score. At the end, the FS method with the smallest sum of ranking scores is ranked at position 1 and so on. One can also divide the final ranking score with the total number of tests and obtain the average rank position. This however does not change the final ranking order. The ranking that we present and conclusions that we derive from it are relative: they are based on the collection of methods that are compared, the collection of criteria used, and the collection of datasets used. Based on our experiments, we observe that the simple DWFS wrapper FS has the best (smallest) average rank across all tests. Yet, for a specific performance metric or a particular dataset, this need not be the case. WEKA is ranked second followed by JMI combined with DWFS. Table 21 presents these ranked positions. Note that in the ranking process all the tested cases equally contribute to the ranking score.

**Table 21:** Sum of ranking points for every method, for every performance metric across all datasets and different classification techniques.

	DWFS	MRMR+DWFS	JMI+DWFS	MRMR	JMI	WEKA	FST3	All features	Correlation-baseline
Ranking for NBC	170	241	201	243	211	142	284	216	193
Ranking for KNN	157	235	207	249	240	235	190	234	245
OVERALL	327 (1^st^)	476 (8^th^)	408 (3^rd^)	492 (9^th^)	451 (6^th^)	377 (2^nd^)	474 (7^th^)	450 (5^th^)	438 (4^th^)

The last row shows the total number of ranking points across all datasets and two classifiers (NBC and KNN) and in parenthesis the final rank position, while in the square brackets the average rank position.

**Fig 4:**
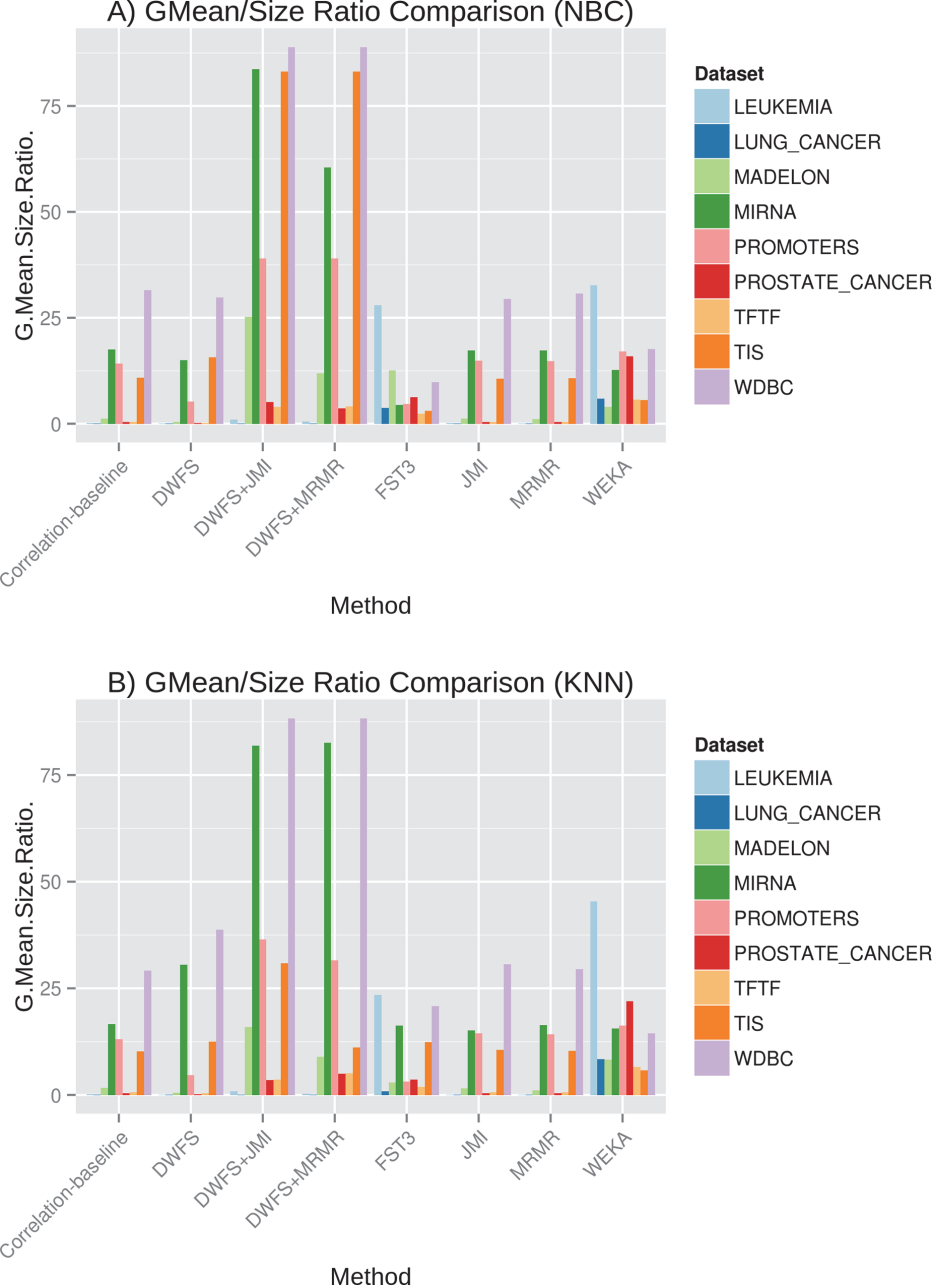
Performance indicator values divided by the absolute number of selected features for all the studied methods and across all the studied datasets. As a performance indicator we use Geometric Mean of Specificity and Sensitivity (GMean). A) Results for NBC classifier. B) Results for KNN

**Effectiveness of selected features and feature subset size reduction**. Next, to provide more insights about the performance of the studied methods we compute the ratio of classification performance over the number of selected features. This ratio measures the effectiveness of the FS process and depicts the maximum classification performance that can be achieved using the reduced number of features selected by an FS method. As performance indicator, we choose GMean since it combines two other important performance metrics (note that other selections were also possible). The results for the NBC and KNN classifiers are presented in Fig. 4. DWFS and its variants achieve the best results in 10 out of 18 tested cases. For the remaining 8 cases (Lung cancer, Leukemia, Prostate cancer and TFTF datasets) WEKA is the best method for both the NBC and KNN classifiers.

In order to quantify the reduction of the number of features we compute the percentage of the extracted features over all features. Tables 22 and 23 present results for the NBC-based wrapper FS and KNN-based wrapper FS, respectively. We observe that DWFS combined with JMI and mRMR leads to the largest feature set size reduction. On average and across all studied datasets, the percentage of the finally selected features for the NBC classifier for DWFS+mRMR and DWFS+JMI is 2.21% and 1.94% of all features, respectively. Regarding the KNN classifier, the percentage of the selected features for DWFS+mRMR and DWFS+JMI, is 2.40% and 2.31% of all features, respectively.

**Table 22:** Feature subset reduction over the original feature set size using NBC-based wrapper FS.

	DWFS	DWFS+MRMR	DWFS+JMI	WEKA	FST3
WDBC	10.67	3.33	3.33	18.00	30.67
Promoters	29.82	3.86	3.86	8.77	27.72
miRNA	12.50	2.92	2.08	13.75	31.25
Prostate cancer	25.84	1.13	0.84	0.26	0.62
Leukemia	32.04	1.90	1.36	0.04	0.04
Lung cancer	39.51	3.15	2.81	0.06	0.08
TFTF	37.87	1.53	1.45	1.11	2.24
TIS	6.91	1.23	1.23	19.26	33.83
Madelon	32.32	0.88	0.48	3.08	0.96
AVERAGE	25.28	2.21	1.94	7.15	14.16

We report the average number of selected features as % of all features.

**Table 23:** Feature subset reduction over the original feature set size using KNN-based wrapper FS.

	DWFS	DWFS+MRMR	DWFS+JMI	WEKA	FST3
WDBC	8.00	3.33	3.33	20.67	14.67
Promoters	31.93	4.56	3.86	8.77	44.91
miRNA	5.73	2.08	2.08	10.83	11.25
Prostate cancer	25.38	0.87	1.22	0.17	1.09
Leukemia	33.67	2.66	1.43	0.03	0.05
Lung cancer	42.13	3.96	4.03	0.04	0.36
TFTF	16.01	1.26	1.75	0.94	3.31
TIS	8.40	1.73	1.98	18.02	8.40
Madelon	33.00	1.16	1.12	2.12	6.16
AVERAGE	22.69	2.40	2.31	6.84	10.02

We report the average number of selected features as % of all features.

**Run time**. In Table 24 and Table 25 we report the run time required for selecting features and evaluating classification performance. From this comparison we exclude mRMR and JMI since they are filtering methods and their run time is much shorter than for any of the wrapper models. We observe that on average and across different datasets, DWFS is the slowest. However, the combination of DWFS wrapper with mRMR reduces significantly the run time and achieves the fastest wrapper setting compared to WEKA and FST3.

**Table 24:** Actual average execution time in seconds for NBC-based wrapper FS and NBC classification.

	DWFS	DWFS+MRMR	DWFS+JMI	WEKA	FST3
WDBC	95.865	87.244	86.929	1.390	0.400
Promoters	94.935	84.663	83.429	1.005	0.800
miRNA	325.707	191.002	177.830	19.491	123.000
Prostate cancer	438.602	97.138	91.955	21.737	21.600
Leukemia	70987.848	197.621	161.578	69.273	15.600
Lung cancer	12867.528	681.727	1203.685	1853.307	14600.600
TFTF	1973.524	215.518	166.770	42.360	56.200
TIS	1048.187	367.765	474.072	211.059	911.800
Madelon	2337.597	181.632	146.899	109.002	3.000
AVERAGE	10018.866	233.812	288.127	258.736	1748.111

**Table 25:** Actual average execution time in seconds for KNN-based wrapper FS and KNN classification.

	DWFS	DWFS+MRMR	DWFS+JMI	WEKA	FST3
WDBC	100.000	88.573	91.502	3.163	2.000
Promoters	91.843	83.981	84.485	1.354	0.800
miRNA	559.724	239.878	235.595	304.241	4273.000
Prostate cancer	400.253	88.260	92.542	20.526	20.600
Leukemia	70331.796	179.031	153.672	67.096	67.800
Lung cancer	10223.792	758.933	1090.058	1897.850	5223.000
TFTF	1054.383	158.576	220.117	167.195	4240.200
TIS	5727.563	19864.425	11747.694	43164.636	14319.000
Madelon	6969.416	284.053	301.029	739.968	14217.800
AVERAGE	10606.530	2416.190	1557.410	5151.781	4707.133

**Fig 5:**
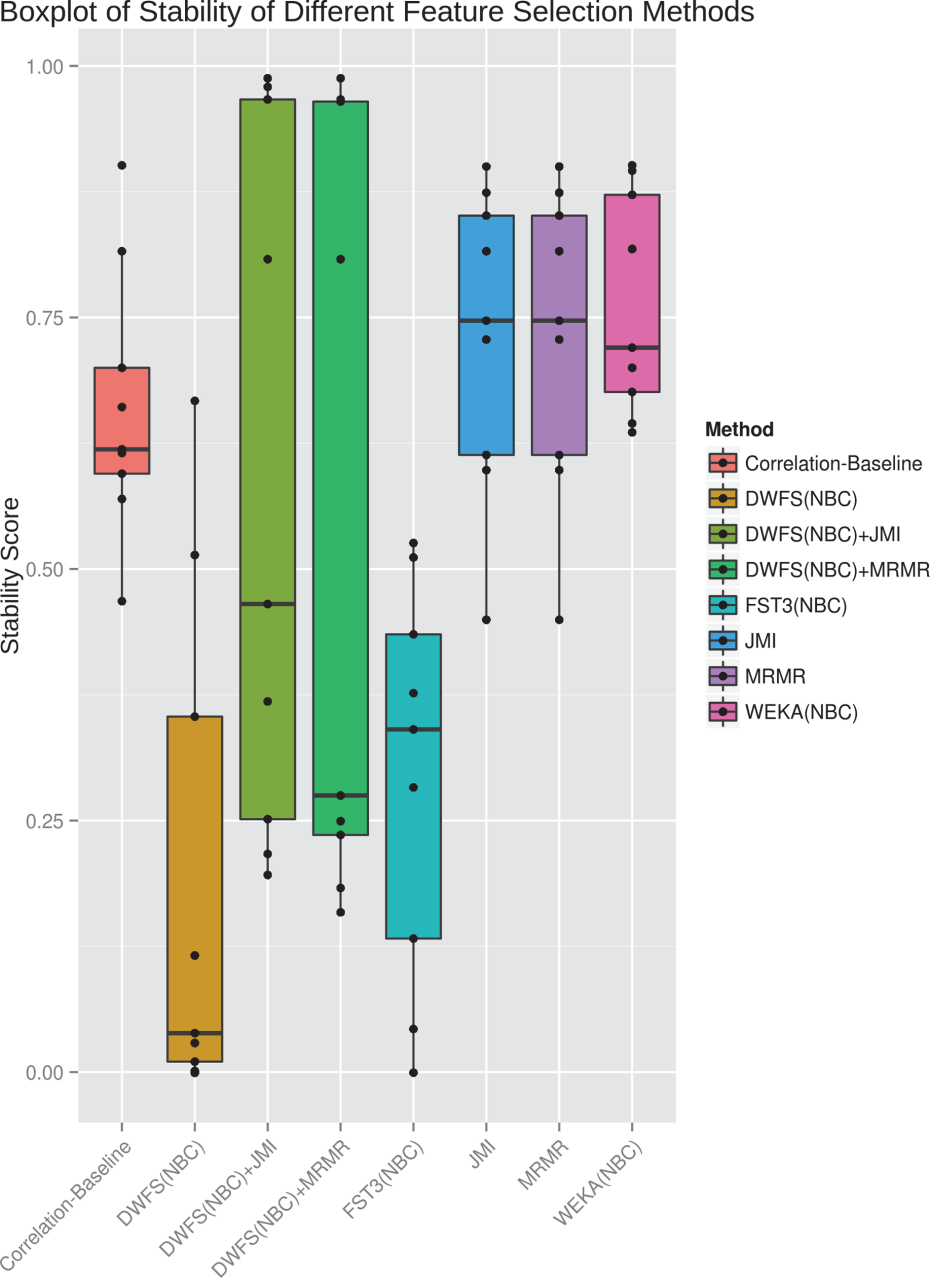
Stability of different FS methods across all datasets using the NBC classifier.

**Stability results**. Stability quantifies the degree of presence of selected features over different splits of the training data. Features that appear more frequently in selected subsets may represent more significant ones for the class prediction, but this factor is not explicitly correlated to classification performance. Note, that most stability metrics are defined for feature subsets of equal size. However, this is not the case for wrappers, where the final selected subsets have different size for every split of data. In our case, we use a definition proposed by Lustgarten et al. [33] (see Equation (8)), which meets our need for measuring stability between feature subsets of different sizes. It should be noted that higher stability may not necessarily reflect a better classification performance. Fig. 5 depicts the results of the comparison analysis using the NBC classifier for all the methods used and across all studied datasets. We observe that filtering approaches are more stable than wrapper-based ones in FS. WEKA with forward and backward search achieves more stable results than the other wrappers. For specific datasets (i.e. WDBC, TIS and Pre-miRNAs), DWFS combined with any of the filters achieves the most stable results.

## Conclusion

In this study we present DWFS, a web-based FS tool based on the wrapper model combined with a randomized search strategy. DWFS offers a variety of options to enhance the optimized FS including incorporation of filtering methods as pre-processing step and many other options for tailoring the objective function according to application requirements. From our extensive experimentation with several benchmark datasets from different biological and biomedical problems we observe that, DWFS integrated in a hybrid setup with a filtering approach for FS is capable of reducing significantly the number of features without sacrificing classification performance. Moreover, using different performance indicators we show that DWFS performs well relative to the existing FS methods. We hope that DWFS will find good use in the analysis of many complex biological and biomedical data complementing other available tools for FS that biologists may use.

## Supporting Information

S1 Table Effects of changing crossover rate on stability of NBC and reduction in the size of selected features. (XLS)

S2 Table Effect of changing mutation rate on stability of NBC and reduction in the size of selected features. (XLS)
